# Predictive imaging for thoracic aortic dissection and rupture: moving beyond diameters

**DOI:** 10.1007/s00330-019-06320-7

**Published:** 2019-07-05

**Authors:** Bouke P. Adriaans, Joachim E. Wildberger, Jos J. M. Westenberg, Hildo J. Lamb, Simon Schalla

**Affiliations:** 1 Department of Radiology and Nuclear Medicine, Maastricht University Medical Center+, P. Debyelaan 25, 6229 HX Maastricht, the Netherlands; 2 Department of Cardiology, Maastricht University Medical Center+, Maastricht, the Netherlands; 3 Cardiovascular Research Institute Maastricht (CARIM), Maastricht University, Maastricht, the Netherlands; 4 Department of Radiology, Leiden University Medical Center, Leiden, the Netherlands

**Keywords:** Aorta, Aortic dissection, Aortic aneurysm, Type A dissection, Aortic rupture

## Abstract

**Abstract:**

Acute aortic syndromes comprise a group of potentially fatal conditions that result from weakening of the aortic vessel wall. Pre-emptive surgical intervention is currently reserved for patients with severe aortic dilatation, although abundant evidence describes the occurrence of dissection and rupture in aortas with diameters below surgical thresholds. Modern imaging techniques (such as hybrid PET-CT and 4D flow MRI) afford the non-invasive assessment of anatomic, hemodynamic, and molecular features of the aorta, and may provide for a more accurate selection of patients who will benefit from preventative surgical intervention. In the current review, we summarize evidence and considerations regarding predictive aortic imaging and highlight evolving imaging modalities that have shown promise to improve risk assessment for the occurrence of dissection and rupture.

**Key Points:**

*• Guidelines for the preventative management of aortic disease depend on maximal vessel diameters, while these have shown to be poor predictors for the occurrence of catastrophic acute aortic events.*

*• Evolving imaging modalities (such as 4D flow MRI and hybrid PET-CT) afford a more comprehensive insight into anatomic, hemodynamic, and molecular features of the aorta and have shown promise to detect vessel wall instability at an early stage.*

## Introduction

“Upon examining the heart, its pericardium was found distended with a quantity of coagulated blood, nearly sufficient to fill a pint cup; the whole heart was so compressed as to prevent any blood contained in the veins from being forced into the auricles; therefore, the ventricles were found absolutely void of blood; and, in the trunk of the aorta, we found a transverse fissure on its inner side, about an inch and a half long, through which some blood had recently passed … ”

In 1760, King George II of Great Britain died unexpectedly while “straining on the toilet,” and so became subject of the first ever case report on acute type A aortic dissection [1]. At autopsy, the King’s personal physician described findings of an intimal vessel wall tear and subsequent cardiac tamponade, which—as we now know—is one of the most feared complications of dissection. Along with pathophysiologically distinct entities like aneurysm rupture and intramural hematoma (IMH), dissection belongs to the spectrum of acute aortic syndromes (AASs). Despite best efforts, these have proven challenging to predict, and annual incidence rates have been stable at approximately 10 per 100,000 over the past decades [2, 3]. Cardiovascular imaging plays a central role in the preventative management of aortic disease, since guidelines traditionally depend on diameter criteria for stratification towards prophylactic surgical intervention [4, 5]. In the current review, we summarize evidence and considerations regarding predictive aortic imaging and highlight modern imaging techniques that have shown promise to improve risk assessment for the occurrence of dissection and rupture.

## Best current practice—aortic diameters

### Normal diameters

**Fig. 1:**
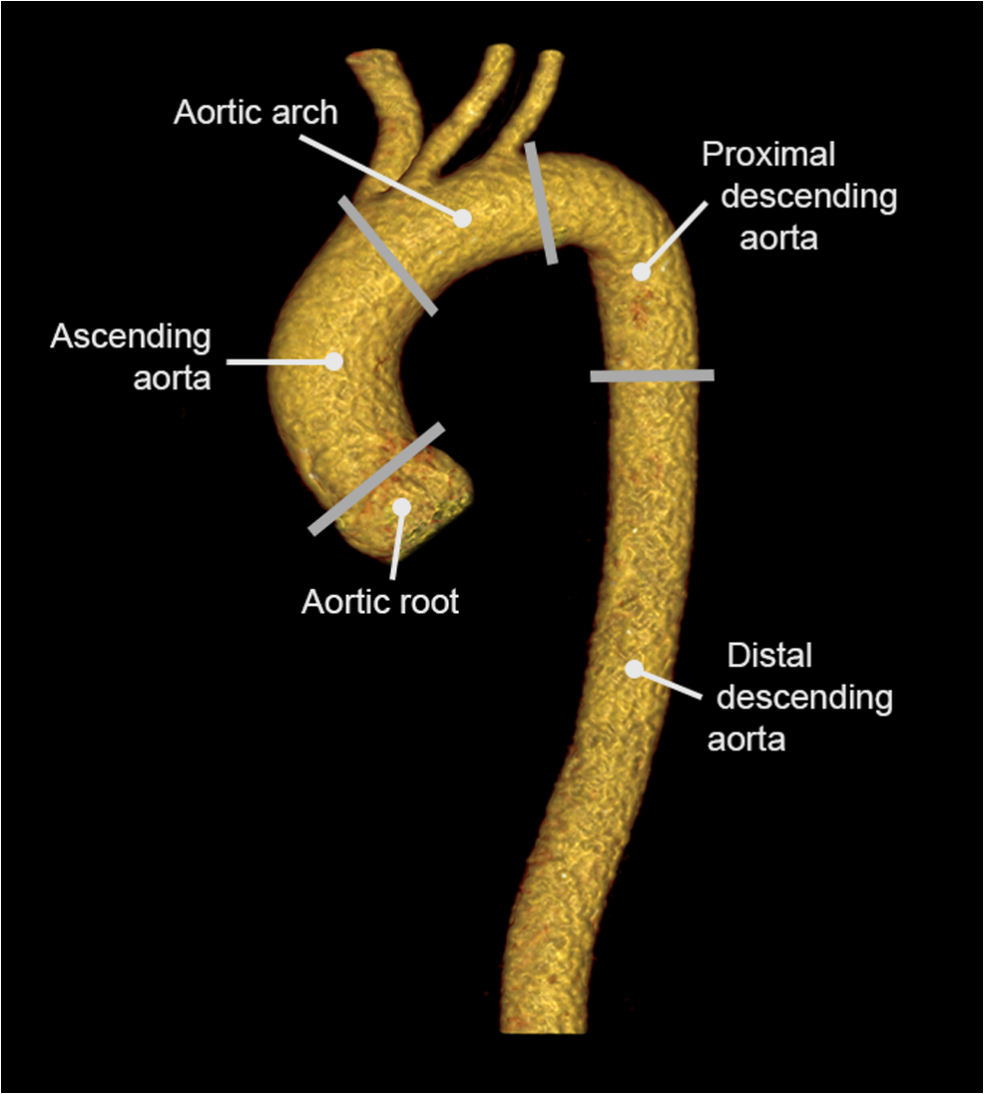
Three-dimensional CT reconstruction of a healthy thoracic aorta. The ascending aorta runs from the sinotubular junction until the first branch vessel (brachiocephalic trunk), while the aortic arch is defined as the segment that contains the three branch vessels. The descending thoracic aorta is divided into two parts: a proximal part (from the left subclavian artery to the level of the pulmonary artery) and a distal part (from the pulmonary artery to the diaphragm)

The aorta is the largest artery in the body and runs from the aortic valve until the abdominal bifurcation. From proximal to distal, it consists of the aortic root, ascending aorta, aortic arch, descending thoracic aorta, and abdominal aorta (Fig. 1). Cross-sectional diameters are influenced by gender, patient habitus, and hypertension, and increase in an indolent manner by approximately 0.1 mm/year [6]. Reference values for the different anatomic segments have been established by multiple imaging modalities, including echocardiography, CT, and MRI [7–9]. Imaging guidelines provide specific measurement recommendations for each of these techniques and emphasize that there exists no standardized method across modalities [10]. Therefore, diameters can vary slightly depending on trigger time (end-systolic vs. end-diastolic) and edge selection (leading edge-to-leading edge vs. inner edge-inner edge vs. outer edge-outer edge). In general, it is stressed that measurements should be performed perpendicular to the aortic centerline (i.e., on double oblique images), and that measurement location and methodology should be specified in order to provide for accurate follow-up in individuals with an indication for repetitive imaging [11–13].

### Thoracic aortic aneurysm

An aneurysm is defined as a localized arterial dilatation of ≥ 2 standard deviations (SDs) above the expected vessel diameter [14]. The underlying pathophysiological mechanisms differ partially for aneurysms at various locations along the aorta. Whereas thoracic aortic aneurysm (TAA) results from excessive degeneration of the medial layer of the vessel wall (also known as cystic medial necrosis), the formation of abdominal aortic aneurysms (AAAs) is mainly associated with atherosclerosis [15]. However, the net result—extensive remodeling of the extracellular matrix of the vessel wall with loss of vascular smooth muscle cells (VSMCs) and elastin content—is similar for all aneurysms [15].

**Fig. 2:**
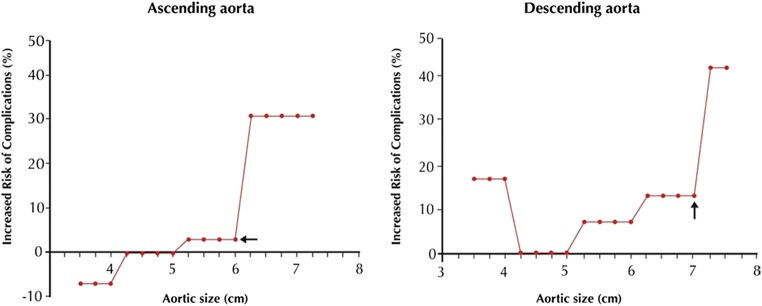
Lifetime probability of dissection or rupture at various sizes of the thoracic aorta. Note the “hinge points” at 6 cm (ascending aorta) and 7 cm (descending thoracic aorta), at which the natural complication risk suddenly escalates. Reprinted from Elefteriades et al, *J Am Coll Cardiol* 2010;55(9):841–857, with permission from Elsevier

Progressive aortic dilatation is a well-acknowledged risk factor for the occurrence of both acute dissection and rupture. While these natural complications are rare in ascending aortic aneurysms of moderate size (yearly rupture or dissection risk of 0.08%, 0.22%, and 0.58% at diameters of 45, 50, and 55 mm, respectively), a sharp step-up in their occurrence—to 6.9% yearly—is observed when the diameter exceeds 60 mm (Fig. 2) [16–18]. In descending thoracic aneurysms, a similar “hinge point” is identified at 70 mm [18]. In order to avoid aneurysm expansion beyond these critical points, the general consensus is to refer patients for pre-emptive surgery at 55 mm (ascending aorta) or 55 to 60 mm (descending thoracic aorta, depending on the eligibility for an endovascular approach) [4, 5, 19]. More frequent surveillance imaging and lower surgical cut-offs apply to patients with connective tissue diseases (such as Marfan syndrome), who are at increased risk for negative outcomes [5]. In these patients, surgery is indicated at diameters ≥ 50 mm, or even ≥ 45 mm in the co-presence of additional risk factors (growth rate > 3 mm/year, familial history of dissection, or severe valvular regurgitation). Patients with bicuspid aortic valve (BAV) and concomitant risk factors are also considered for surgery at a lower than normal threshold (≥ 50 mm), although evidence for this approach is lacking [4, 5, 20]. Admittedly, BAV is overrepresented in large dissection cohorts, but the higher number of aortic events in this patient group cannot be seen independent from its increased TAA prevalence [17, 21]. At any given TAA diameter, the yearly aortic complication risk for bicuspid and tricuspid valves has shown to be comparable [22].

### Aortic size paradox

Despite the evident link between TAA formation and unfavorable outcomes, the vast majority of dissections occur in aortas with diameters below the threshold for preventative surgery—the so-called aortic size paradox. Retrospective studies have shown that only 30–41% of patients with type A and 18% of patients with type B dissection had diameters ≥ 5.5 cm at the time of presentation [23–26]. Since the aorta dilates by about one-third of its size directly after dissection onset, the number of events that could have been prevented by current diameter cut-offs is probably even lower [27–29]. Nevertheless, it is questionable if lowering the thresholds for surgical intervention would bear a long-term mortality benefit. Given the large population at risk, it would more likely expose a considerable number of patients with smaller TAAs—and thus, minimal yearly risk of natural complications—to the 3.7–8.3% mortality risk associated with elective surgery [30]. In conclusion, it could be stated that the aortic diameter predicts rupture and dissection on a populational level, but is an insufficient parameter to identify individuals at risk. For this reason, recent research interests have shifted to the deciphering of additional risk factors for AAS, in an attempt to enhance personalized risk assessment and clinical decision-making.

## Modern perspectives—moving beyond diameters

### Aortic elongation and volume

**Fig. 3:**
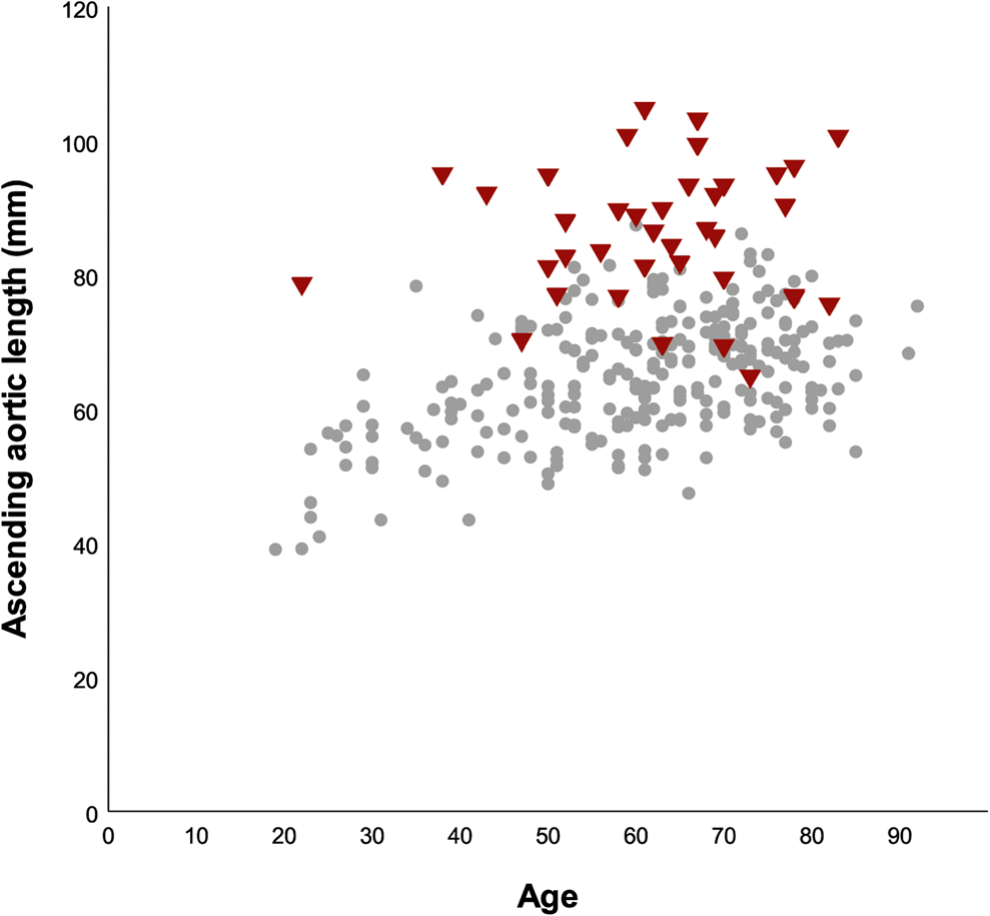
Scatter plot depicting the length of the ascending aorta in patients with acute type A dissection (red) and healthy controls (gray). In the majority of dissection patients, the aorta was evidently lengthened (mean difference of 2.0 cm when compared with propensity-matched healthy controls). Adapted by permission from BMJ Publishing Group Limited, from Heuts et al, Aortic elongation part II: the risk of acute type A aortic dissection, *Heart* 2018;104:1778–1782

One drawback of maximal diameter measurements is that they do not adequately represent the three-dimensional process of aortic growth. Aneurysm lengthening and cylindrical deformation are two scenarios of positive remodeling that are not necessarily accompanied by an increase of maximal diameters [31]. Since more advanced acquisition and post-processing techniques are required to assess the three-dimensional geometry of the aorta, data on its length and volume are relatively scarce. Similar to its diameters, normal aortic length has shown to increase with age [32, 33]. With the vessel being confined within the thoracic cavity, this lengthening process naturally causes the artery to become more tortuous [32]. Driven by observations that the intimal entry tear runs in the transverse direction in the majority of cases (i.e., results from disruptive stretch in the longitudinal direction), recent studies have investigated the role of excessive elongation in the pathophysiology of dissection [34, 35]. They found that increased vessel curvature significantly elevates the forces acting on the aortic wall and that vessel length serves as an independent risk factor for the occurrence of both type A and type B dissection (Fig. 3) [36–39].

To date, the added value of volume measurements in the management of thoracic aortic disease is not fully understood. Several AAA studies have suggested improved sensitivity for detection of aneurysm growth by reporting that substantial volume expansion can occur even while the maximal diameter remains stable [40–43]. This finding is important, since it implicates (rapid) growth in regions proximally or distally from the widest portion of the aneurysm sac. Volumetry has also shown improved intra- and inter-observer variability when compared with diameter measurements, which is another argument in favor of its use in clinical aneurysm follow-up [44]. Although no causal link with adverse outcomes has yet been established, the cited studies have demonstrated that aortic length and volume can be measured on routinely obtained CT using commercially available software packages. This widespread availability makes them feasible predictive parameters for use in a clinical setting, although longitudinal—and, preferably, prospective—studies are now required as the next step in their validation process.

### Aortic hemodynamics

The role of hemodynamics in the pathogenesis of aortopathy has been subject of a long-standing debate. Especially in BAV patients, who exhibit enlarged aortic diameters even in the absence of valvular dysfunction, there has been controversy regarding the origin of aneurysm formation [45]. Two theories explain the accelerated aortic growth rates and high prevalence of TAA in this patient group: (i) a genetic theory, which rests on evidence that BAV is a congenital condition with considerable genetic heterogeneity and (ii) a hemodynamic theory, in which abnormal flow patterns and turbulence cause elevated wall stress and subsequent vessel remodeling [46]. Several genes (such as *NOTCH1*, *ACTA2*, and *GATA5*) have been associated with abnormal development of the aortic valve [46, 47]. Given that the aortic cusps and the medial layer of the ascending aorta are embryologically linked (i.e., both originate from the neural crest), it is conceivable that the genes responsible for BAV formation can also affect the development of the aortic vessel wall. Other arguments in support of the genetic theory include observations that BAV aortopathy is not uncommon in children and adolescents, and that progressive diameter increase can occur even after replacement of the aortic valve [48, 49]. Initially, the histologic presence of cystic medial necrosis was also presented as an argument for disease inheritability, as this was believed to resemble findings in those with Marfan syndrome [50]. However, later work has demonstrated that medial degeneration is a common feature of all TAAs and dissected aortas, regardless of their underlying etiology [15, 51].

**Fig. 4:**
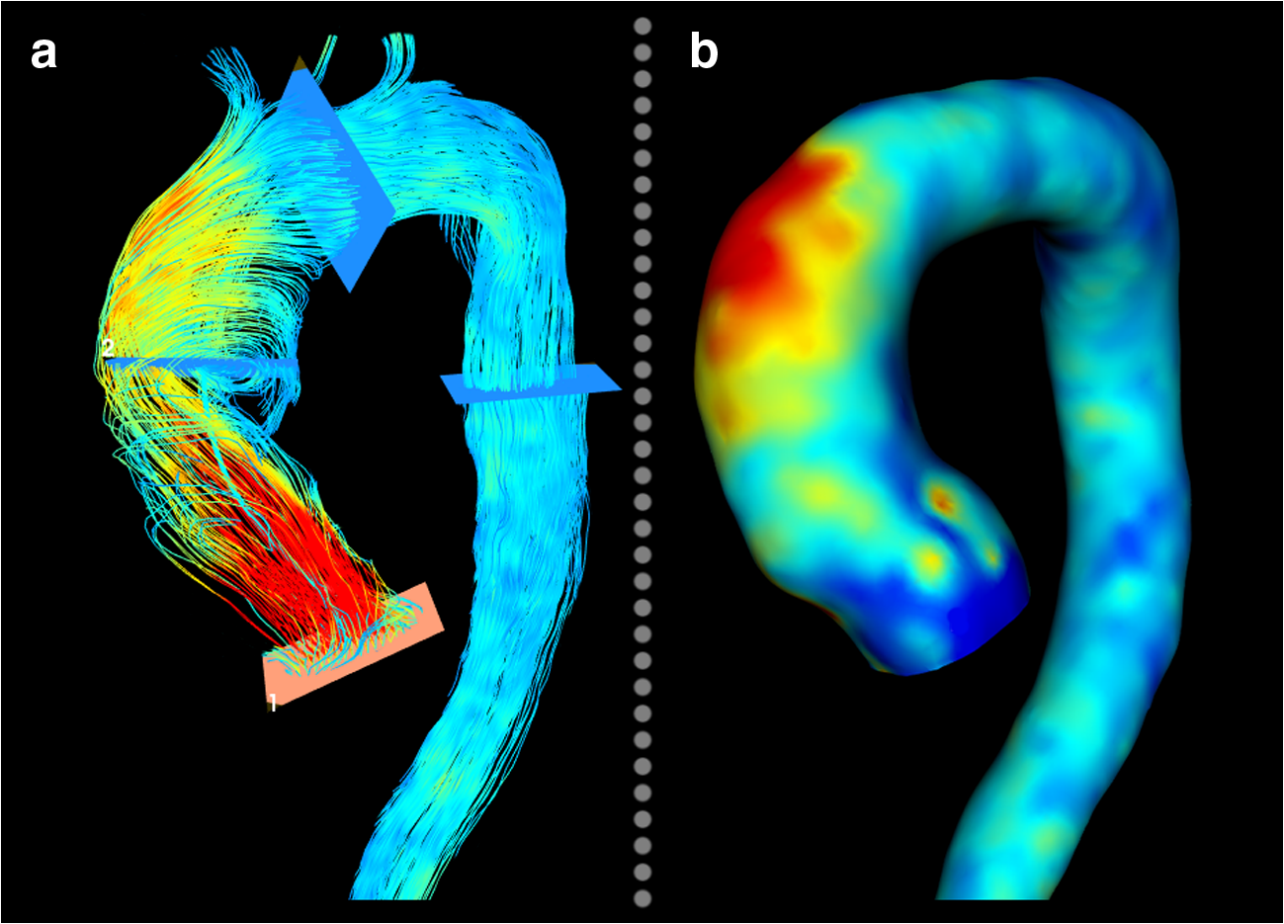
**a** Streamline visualization of a BAV patient, showing a pronounced eccentric and helical flow pattern. **b** The adjacent WSS map showing elevated wall stress in the greater curvature of the aorta, at the location of impingement between the eccentric jet and the vessel wall

**Fig. 5:**
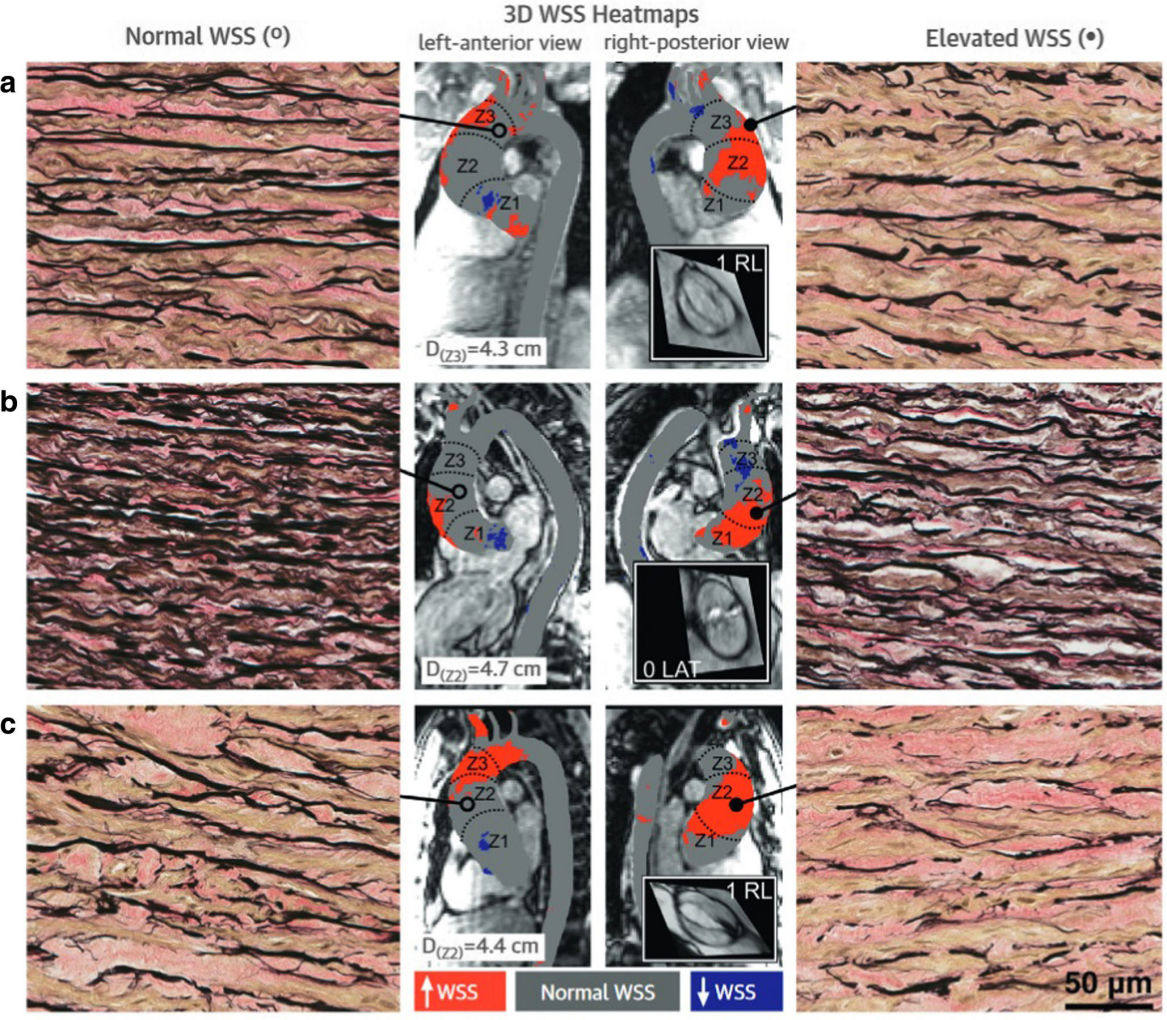
Aortic wall specimens of regions with normal WSS (left panels) and high WSS (right panels) in three (**a**–**c**) patients with BAV aortopathy (× 40 magnification). Note the decreased number of elastin fibers (black) in the context of elevated WSS. Center panel: 4D flow MRI–based maps depicting areas with increased (red) and depressed (blue) WSS. Reprinted from Guzzardi et al, Valve-related hemodynamics mediate human bicuspid aortopahy, *J Am Coll Cardiol* 2015;66:892–900, with permission from Elsevier

Over the past few years, the hemodynamic theory has become increasingly popular, along with the evolvement of functional imaging modalities that afford a more comprehensive insight into aortic hemodynamics. In flow MRI, phase-contrast (PC) techniques are used to generate image contrast between moving protons (such as in blood) and stationary protons (most soft tissues). The underlying concept is based on the feature of protons to accumulate an MRI phase shift that is proportional to the speed at which they move along a magnetic gradient field. Phase-contrast MRI is traditionally performed using a manually positioned two-dimensional (2D) acquisition that encodes velocity in one principal direction. This approach generates time-resolved velocity maps, which can be used to quantify flow rates and velocities of blood moving through the imaging slice. As such, it enables evaluation of a broad spectrum of cardiovascular diseases, including assessment of shunt fractions and valvular regurgitant volumes. Over recent years, methodological advances have facilitated the acquisition of time-resolved, three-dimensional, three-directionally encoded velocity data. This technique, commonly known as 4D flow MRI, affords a uniquely detailed flow visualization within the heart and large vessels and allows post hoc flow quantification at any location within an acquired volume [52]. Furthermore, the obtained velocity data can be used for estimation of various flow-derived hemodynamic parameters, including wall shear stress (WSS) and normalized flow displacement. In BAV patients, 4D flow MRI has revealed markedly eccentric and helical flow with highest flow velocities and WSS located along the aortic vessel wall (Fig. 4) [53, 54]. For each BAV cusp fusion type, a typical WSS distribution pattern with elevated shear stress at the location of impingement between the flow jet and vessel wall was identified [55]. Subsequently, raphe-specific WSS patterns have shown to correspond with the phenotype of BAV aortopathy (i.e., left-right coronary cusp fusion leads to sole dilatation of the tubular ascending aorta, whereas a raphe between the right- and non-coronary cusps causes more diffuse dilatation with involvement of the aortic root, tubular ascending aorta, and aortic arch) [56, 57]. In a recent contribution, Guzzardi et al have also demonstrated a more direct association between WSS and histologic changes of the aortic vessel wall [58]. In their study, BAV patients who were scheduled for ascending aortic replacement underwent pre-operative WSS mapping. During surgery, paired tissue samples of aortic regions with normal and elevated WSS were collected to show that increased WSS was associated with dysregulation of the extracellular matrix and degeneration of elastin fibers (Fig. 5).

Flow displacement is another 4D flow MRI parameter that has shown potential to predict progression of aortic disease. It provides a quantitative measure of flow eccentricity by calculating the distance between the location of peak systolic flow and vessel center in a 2D imaging plane [59]. Like WSS, the degree and direction of flow displacement depend on the underlying aortic valve phenotype [56]. As yet, it is the only 4D flow MRI parameter that has been associated with aortic growth in a (small) longitudinal cohort study. In this study (mean follow-up duration, over 4 years), BAV patients with eccentric aortic flow exhibited faster diameter growth than those with laminar flow profiles (1.2 mm/year vs. 0.3 mm/year, respectively) [60]. Larger longitudinal studies that aim to acquire the clinical relevance of various 4D flow MRI parameters are currently ongoing.

### Vessel wall inflammation

**Fig. 6:**
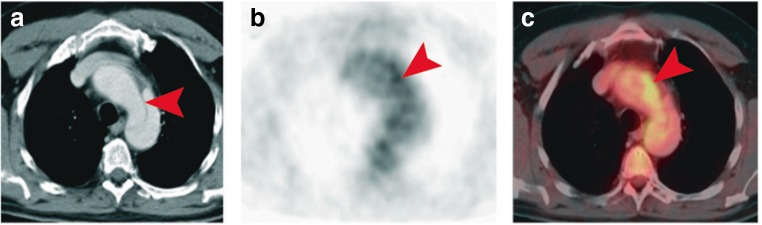
**a** Contrast-enhanced CT depicting type B dissection (arrowhead) in a patient with acute chest pain. **b**, **c** Adjacent PET and PET-CT examinations revealed regions of elevated FDG uptake in the dissected vessel wall (arrowheads). Adapted by permission from BMJ Publishing Group Limited, from Kuehl et al, *Heart* 2008;94:1472–1477

Immunohistochemical studies have reported extensive inflammatory activity within the aneurysmatic vessel wall [15]. Whereas the presence of lymphocytes and activated macrophages is rare in a healthy aorta, elevated numbers of CD3^+^ and CD68^+^ cells were found throughout the medial layer of TAAs [51]. Of note, infiltration of these cells has shown to be even more pronounced in the dissected aorta, raising the question whether the degree of inflammation can be used to discriminate between low- and high-risk patients [51]. Since leukocytes demand glucose for their accelerated metabolic processes, areas of increased inflammatory activity can be detected using positron emission tomography (PET). This imaging modality depends on measurements of radioactivity emitted after administration of a radioactive tracer, most commonly the glucose derivative 18-fluoro-2-deoxyglucose (FDG; > 95% of all PET examinations). FDG is transported into cells by glucose transporters and becomes phosphorylated to form FDG-6-phosphate. Unlike glucose-6-phosphate, FDG-6-phosphate is not further metabolized along the glycolytic pathway and becomes trapped within the cell in a concentration that is in proportion to that cells’ glucose consumption. In animal experiments, FDG avidity has shown to be positively correlated with macrophage content of the arterial vessel wall [61, 62]. A similar association has been observed in vivo in patients with abdominal aneurysms [63, 64]. Furthermore, the AAA study by Reeps et al reported FDG uptake to be associated with a decrease in collagen content and VSMCs—factors that determine the stability of the aortic wall. However, despite these promising results, studies that sought to investigate the use of FDG-PET for prediction of AAA expansion and rupture have reported conflicting results. Whereas two prospective studies have demonstrated elevated tracer uptake to be associated with disease progression, others could not establish such a relationship [65–70]. As yet, only one study has investigated the use of PET-CT in thoracic aortic disease. In a small sample of hemodynamically stable patients with various acute aortic conditions (e.g., type B dissection, penetrating ulcer, or IMH), this longitudinal study showed increased FDG uptake to be associated with the risk of disease progression (Fig. 6) [71]. Of the 11 included patients with increased FDG uptake, nine (82%) showed progression of wall pathology under conservative treatment requiring emergency medical intervention, and three of these nine patients died. In contrast, only 10 of the 22 (45%) PET-negative patients experienced disease progression (two deaths).

In conclusion, no clear relation between FDG-PET and clinical outcomes in thoracic and abdominal aneurysm patients has yet been demonstrated, although it should be noted that the inconsistency between studies partly relates to their small and heterogeneous study populations. Prospective studies with larger patient cohorts are warranted to assess the actual clinical value of FDG-PET. Also, future work could address the clinical value of novel radiotracers that have shown potential to target other processes within the inflammatory pathway of aneurysmal disease, such as expression of matrix metalloproteases (MMPSense 680), active vascular calcification (18F-NaF), and chemokine receptor 4 expression (68-Ga-pentixafor) [72–74]. The recent integration of PET and MRI into PET/MRI scanners has afforded the synchronized evaluation of anatomic, physiologic, and molecular imaging features, and could serve as a promising imaging platform in the future.

## Conclusions

Excessive aortic dilatation (diameter ≥ 5.5 cm) poses a significant risk for the occurrence of acute aortic events, and patients with aneurysms beyond this size should be referred for prophylactic surgical intervention. However, the majority of acute aortic events occur in aortas with diameters below surgical thresholds. Therefore, these patients would not have qualified for preventative surgery, even when screened appropriately prior to event onset. Several modern imaging techniques (such as hybrid PET-CT and 4D flow MRI) have shown promise to detect vessel wall instability at an early stage. Studies that aim to demonstrate a causal link with the occurrence of acute aortic syndromes are currently ongoing and could lead to the integration of these techniques into clinical practice guidelines.

## Abbreviations

AAA: Abdominal aortic aneurysm
AAS: Acute aortic syndrome
BAV: Bicuspid aortic valve
IMH: Intramural hematoma
PC: Phase contrast
SD: Standard deviation
TAA: Thoracic aortic aneurysm
VSMC: Vascular smooth muscle cell
WSS: Wall shear stress
